# Preliminary Investigation on Simvastatin-Loaded Polymeric Micelles in View of the Treatment of the Back of the Eye

**DOI:** 10.3390/pharmaceutics13060855

**Published:** 2021-06-09

**Authors:** Silvia Pescina, Fabio Sonvico, Adryana Clementino, Cristina Padula, Patrizia Santi, Sara Nicoli

**Affiliations:** Department of Food and Drug, University of Parma, Parco Area delle Scienze 27/a, 43124 Parma, Italy; fabio.sonvico@unipr.it (F.S.); adryana.rochaclementino@studenti.unipr.it (A.C.); cristina.padula@unipr.it (C.P.); patrizia.santi@unipr.it (P.S.); sara.nicoli@unipr.it (S.N.)

**Keywords:** statins, simvastatin, ocular delivery, TPGS, polymeric micelles, trans-scleral, conjunctiva, ex vivo

## Abstract

There is increasing consensus in considering statins beneficial for age-related macular degeneration and in general, for immune and inflammatory mediated diseases affecting the posterior segment of the eye. However, all available data relate to oral administration, and safety and effectiveness of statins directly administered to the eye are not yet known, despite their ophthalmic administration could be beneficial. The aim was the development and the characterization of polymeric micelles based on TPGS or TPGS/poloxamer 407 to increase simvastatin solubility and stability and to enhance the delivery of the drug to the posterior segment of the eye via trans-scleral permeation. Simvastatin was chosen as a model statin and its active hydroxy acid metabolite was investigated as well. Results demonstrated that polymeric micelles increased simvastatin solubility at least 30-fold and particularly TPGS/poloxamer 407 mixed micelles, successfully stabilized simvastatin over time, preventing the hydrolysis when stored for 1 month at 4 °C. Furthermore, both TPGS (1.3 mPas) and mixed micelles (33.2 mPas) showed low viscosity, suitable for periocular administration. TPGS micelles resulted the best performing in delivery simvastatin either across conjunctiva or sclera in ex vivo porcine models. The data pave the way for a future viable ocular administration of statins.

## 1. Introduction

Age-related macular degeneration (AMD) represents one of the main conditions responsible for vision impairment in the adult population and the last World Health Organization forecast indicates 196 million people were affected by AMD in 2020 worldwide [1]. In AMD, the progressive loss of photoreceptors located in the macula, a small area in central retina, is responsible for vision reduction and eventually blindness. 

The etiopathology of the disease is still unclear and two variants of AMD may occur: the most frequent is a non-neovascular condition, also known as the dry form, while the neovascular type, is called wet form. The dry form is characterized by the progressive development of drusen, i.e., extracellular debris deposits between Bruch’s layer and basal lamina of the retinal pigmented epithelium (RPE) and between RPE and retina (subretinal drusenoid deposits), responsible for a progressive and, later on, irreversible retinal damage [2]. Drusen consist of proteins, such as apolipoprotein E, amyloid P component, complement 5c among others [3], and lipids, comprising both free and esterified cholesterol [4]. The dry form may evolve into the neovascular type, which shows a quick retinal angiogenesis and can benefit from anti-VEGF agents, like bevacizumab, ranibizumab or pegaptanib. Unfortunately, apart from the ongoing evaluation of embryonic stem cells [5], no effective treatments are yet available for the dry form. Therefore, the presence of a lipidic matrix, and particularly of cholesterol, explains the growing interest in lipid-lowering agents, like statins, as possible tool in reducing and evenly preventing the progression of degenerative mechanisms [6,7]. In a 2016 clinical study, high dose oral atorvastatin was administered to AMD patients, showing softening of drusenoid deposits. Following a therapeutic protocol consisting of 80 mg/day oral atorvastatin, after one year, both a regression of drusen deposits and an improvement of visual acuity were demonstrated in treated patients [8]. Furthermore, no progression of the disease toward a neovascular form was detected: based on that, the authors hypothesized that an inhibition of hydroxy methylglutaryl CoA (HMGCoA) reductase at retinal level was responsible for the effects observed. A slow-down of the progression of AMD was also highlighted using simvastatin 40 mg/day [9] and was further confirmed by other studies [10,11]. Recently, Mast and co-authors demonstrated in experiments in vivo on mice that simvastatin reduces cholesterol levels inside retina by inhibiting cholesterol biosynthesis and, at the same time, by inducing the overexpression of one of the receptors involved in the clearance of oxidized low-density lipoproteins from Bruch’s membrane [12]. Although data were collected in animals, authors suggested that these results could be considered relevant also for humans and that statins deserve to be investigated for the treatment of AMD. Furthermore, simvastatin showed the ability to protect photoreceptors both in ex vivo (human retinal explants) and in vivo (mouse) models [13].

The complex homeostasis of chorio-retinal cholesterol [14] is presumably not the only factor related to AMD, that is generally considered a multifactorial disease, also involving inflammatory and immune processes [15]. Once again, statins could be beneficial as a consequence of their anti-inflammatory and immunomodulatory characteristics [16]. Indeed, these pleiotropic properties have been responsible for a rising interest in the last two decades in the use of statins for the treatment of ophthalmic diseases. Particularly, orally administered statins in humans reduces retinal inflammation [17], decreases the risk of open-angle glaucoma [18] and prevents non-infectious uveitis [19]. Another study carried on hypercholesterolemic rabbits, demonstrated the ability of orally administered low dosage statins in reducing hypercholesterolemia-associated choroidal injury [20]. Moreover, a cohort study suggests that the systemic administration of statins may prevent, to some extent, postoperative complications of vitreoretinal surgery [21].

However, despite many studies carried out during the last years, the effect of statins on the eye after oral administration is still controversial, since a complete lack of efficacy, particularly in the case of AMD [22,23] and/or side effects like blurred vision and vision impairment, especially associated with the use of atorvastatin [24], were reported. This uncertainty on the role of statins can be explained by the genetic component of some ocular diseases [8], as well as by a wide heterogeneity of clinical protocols in terms of statin selected (simvastatin, atorvastatin, fluvastatin, pravastatin), dosage, therapeutic target, study outcomes and duration, number of participants and administration route [6]. As already highlighted by other researchers, there is an important lack of knowledge on the ophthalmic administration of statins [21]. In fact, to our knowledge, only one example of statin-based ophthalmic formulation is described in the literature, that is the successful use of atorvastatin solution topically administered in patients for the treatment of dry eye blepharitis [25]. The ocular administration, although very challenging, could avoid muscle and liver side effects related to the systemic absorption of high doses of statins [26]. Hence, the development of a statin-based formulation intended for ophthalmic administration and targeting the back of the eye would be extremely interesting. Very recently, topical formulation consisted of atorvastatin-loaded solid lipid nanoparticles for the treatment of AMD [27] and atorvastatin-eluting contact lens addressing the ocular surface diseases [28] were proposed.

**Figure 1:**
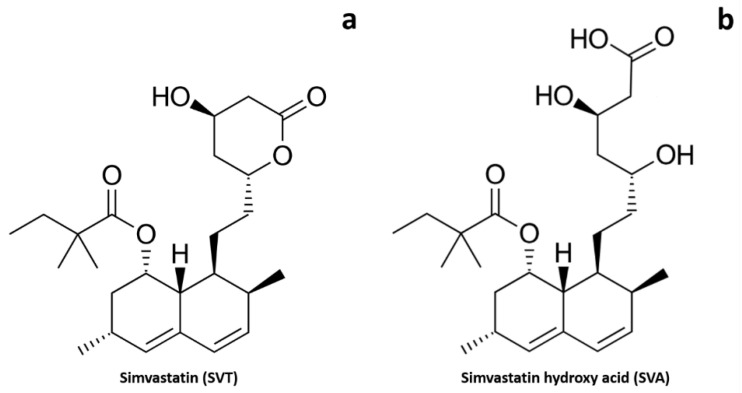
Chemical structures of simvastatin (**a**) and simvastatin hydroxy acid (**b**).

In the present work, we proposed the development of simvastatin-loaded polymeric micelles for targeting the back of the eye via the trans-scleral route, in view of the treatment of dry-AMD and, more in general, of inflammatory condition affecting the posterior segment. Being simvastatin (SVT, Figure 1a) a well-known and widely used statin, it was chosen as reference compound; given the low water solubility, polymeric micelles were selected as vehicle. Polymeric micelles are very versatile colloidal systems and represent an efficient strategy to deliver hydrophobic drug to the eye [29,30], and have been previously proposed as stabilizing system for simvastatin oral administration [31]. In precedent studies, we prepared polymeric micelles using a water-soluble derivative of vitamin E (tocopherol polyethylene glycol succinate, TPGS) and an amphiphilic tri-block copolymer, poloxamer 407. Both excipients are biocompatible and, in addition, TPGS can release antioxidants such as vitamin E and vitamin E succinate in the presence of esterase in vivo [32]. After the in vitro characterization, micelles were tested ex vivo in porcine ocular tissues by quantifying the amount of the pro-drug simvastatin (SVT) and its active metabolite simvastatin hydroxy acid (SVA, Figure 1b) retained in and permeated across the ocular tissues. The role of choroidal melanin in SVT diffusion was also investigated.

## 2. Materials and Methods

### 2.1. Materials

The vitamin E-derived surfactant tocopherol polyethylene glycolsuccinate (TPGS, MW approx. 1513 g/mol) was a kind gift of PMC ISOCHEM (Vert-Le-Petit, France) and poloxamer 407 (P407, MW approx. 12,600 g/mol; Lutrol^®^ F127) was a gift from BASF (Ludwigshafen, Germany). Simvastatin (SVT; [(1S,3R,7S,8S,8aR)-8-[2-[(2R,4R)-4-hydroxy-6-oxooxan-2-yl]ethyl]-3,7-dimethyl-1,2,3,7,8,8a-hexahydronaphthalen-1-yl]- 2,2-dimethylbutanoate; MW 418.57 g/mol; LogD_7_._4_ 1.6 [33]; predicted pk_a_ 13.5 [34]; practically insoluble in water) was from Polichimica s.r.l. (Bologna, Italy).

Simvastatin hydroxy acid (SVA; (3R,5R)-7-[(1S,2S,6R,8S,8aR)-8-(2,2-dimethylbutanoyloxy)-2,6-dimethyl-1,2,6,7,8,8a-hexahydronaphthalen-1-yl]-3,5-dihydroxyheptanoic acid; MW 436.58 g/mol; LogD_7_._4_ 1.81 [35]; predicted pKa 4.3 [34]), also known as tenivastatin, was prepared as previously described [36,37]. Briefly, 40 mg of SVT were dissolved in 1 mL of ethanol; then, 1.5 mL of 1 M NaOH were added and the solution kept at 50 °C for two hours. After cooling, pH was adjusted to 7.2 using HCl, then pure water was added to reach the final concentration of 4.0 mg/mL. SVA solution was finally stored at −20 °C.

Saline solution (N; 9 g/L NaCl) and phosphate buffered saline (PBS; composition: 0.19 g/L KH_2_PO_4_, 2.37 g/L Na_2_HPO_4_, 8.8 g/L NaCl; pH 7.4 by adding 85% H_3_PO_4_) were prepared using purified water (Purelab^®^ Pulse, Elga Veolia, High Wycombe, UK). All chemicals used were of analytical grade.

### 2.2. Micelle Preparations

TPGS and TPGS/P407 mixed micelles were prepared as described earlier [32].

#### 2.2.1. TPGS Micelles (TN)

TPGS was solubilized in saline solution (N) to obtain a 20 mM (3% *w*/*v*) blank TPGS micellar solutions (TN). Then, 1 mg SVT was added to 1 mL TN, and left under magnetic stirring overnight, to obtain simvastatin-loaded polymeric micelles (TN_SVT_). As alternative loading method, SVT was added once dissolved in ethanol (see Supplementary Materials, Table S1). 

#### 2.2.2. TPGS/P407 Mixed Micelles (MN)

Poloxamer 407 solutions were prepared by dissolving poloxamer 407 in saline solution (N) to a final concentration of 20 mM (corresponding to 25.2% *w*/*v*). Mixed micelles, MN, were obtained by mixing in 1:1 ratio 20 mM P407 to 20 mM TPGS solutions (final P407 and TPGS concentration, 10 mM each). Simvastatin was loaded by directly adding 1 mg of powder to 1 mL of mixed micelles (MN_SVT_), then kept overnight under magnetic stirring. As alternative, SVT was loaded as ethanolic solution (see Supplementary Materials, Table S1).

### 2.3. SVT Solubility in Micelles

SVT powder was added in excess to 1 mL of TN or MN and left overnight at room temperature under magnetic stirring. Suspension was then quantitatively transferred into a polypropylene microtube and repeatedly centrifuged at 12,000 rpm for 10 min to obtain a completely clear solution. The supernatants were diluted, and the concentration of SVT was determined using HPLC/UV-Vis (see Section 2.10). Each condition was tested in triplicate. Furthermore, SVT solubility in saline solution was ascertained following the same procedure.

### 2.4. Viscosity

Viscosity of blank micellar formulations (TN and MN) was measured using an Ares rheometer (TA Instruments, New Castle, DE, USA), equipped with an RSI Orchestrator software (TA Instruments). Measurement was done at 25 °C under strain-controlled conditions, using a Couette geometry. The rotation took place in clockwise direction and the measure rate was set from 0.02 to 300 1/s.

### 2.5. Dynamic Light Scattering 

Dynamic Light Scattering (DLS) was used for both blank (TN, MN) and SVT loaded micelles (TN_SVT_, MN_SVT_) to determine the mean size as well as the polydispersity index (PdI) at time zero, after 48h and 1 month storage at room temperature. Samples were analysed without any dilution using a Zetasizer Nano ZS (Malvern Panalytical Ltd., Malvern, UK) at 25 °C, with incidence angle of 173° (refractive index 1.33; viscosity 0.8872 cP). Size distribution was reported as z-average calculated using intensity.

### 2.6. Stability

The stability of SVT in micelles stored at +4 °C and +25 °C was evaluated after 24 h, 48 h and 1 month, by quantifying the amount of SVT and SVA using HPLC. Samples were diluted 1:100 in the mobile phase (composed of 65% acetonitrile and 35% 25 mM phosphate pH 4.5; see Section 2.10).

### 2.7. Ocular Tissues Preparation

Conjunctiva, sclera (S), and trilayer sclera-choroid-Bruch’s membrane (SCh) were isolated from fresh porcine eyes, obtained from a local slaughterhouse (Macello Annoni S.p.A., Busseto, Italy); breed: Landrace and Large White, both female and male animals; age: 10–11 months; weight: 145–190 kg). After enucleation, eyebulbs were soaked in PBS at +4 °C and transferred to the laboratory: conjunctival tissue, corresponding to the forniceal portion, was isolated from the inferior part of the eye bulb and used within 4 h from enucleation [38]. S and SCh were separated once the connective tissue surrounding the bulb was removed, the anterior and posterior segments were divided, and vitreous body, as well as retina were eliminated, as previously described [39]. Both pigmented and not-pigmented choroids were used [40].

### 2.8. Validation of SVT and SVA Extraction Method from Sclera and Choroid

In order to validate SVT and SVA quantification from the ocular membranes, 0.6 cm^2^ tissue samples, obtained by punching the plain thawed sclera, were put inside a polypropylene microtube; 15 μL of a 5 mg/mL SVT solution in ethanol were added (SVT total amount 75 μg). After 1 h, allowing for ethanol evaporation and drug penetration in the tissue, 0.5 mL of extraction mixture composed of 65% acetonitrile and 35% 25 mM phosphate pH 4.5 (mobile phase: see Section 2.10 HPLC/UV-Vis analysis) were added and vortexed every 15 min, for one hour. Tissue was then transferred into a new polypropylene microtube and the above procedure was repeated. Then, the second extractive aliquot was combined to the previously collected and analysed by HPLC/UV-Vis for SVT and SVA quantification. The above-mentioned method was validated for specificity and recovery. The total recovery was 99.36 ± 1.9% and consisted almost exclusively of SVT, since SVA recovered was under the limit of quantification (LOQ; see Section 2.10 HPLC/UV-Vis analysis).

A similar procedure was followed for thawed choroid (Ch): in this case, 15 μL of a 0.5 mg/mL SVT solution in ethanol were added (SVT total amount 7.5 μg). After the evaporation of ethanol, SVT was recovered using 0.5 mL of mobile phase. The extraction lasted 1 h, during which sample was vortexed every 15 min. The amount of SVT recovered was 100.4 ± 2.8%, while no SVA was detected.

### 2.9. Ex Vivo Experiments

#### 2.9.1. Trans-Conjunctival Permeation

For the trans-conjunctival experiments [38], Franz cells with a permeation area of 0.2 cm^2^ were used. The donor compartment was alternatively filled with 0.25 mL of 1 mg/mL SVT in TN_SVT_ (pH 6.78 ± 0.05), MN_SVT_ (pH 6.58 ± 0.02) and 1 mg/mL SVA saline solution (pH 6.75 ± 0.05); the receiving phase, consisted of TPGS 0.5 mM in PBS pH 7.4, was previously degassed and maintained at 37 °C and under magnetic stirring to avoid any boundary layer effect. Each experiment lasted 5 h and at every hour, the receiving phase was sampled and analyzed for quantifying both SVT and SVA. The amount of permeated compound (µg/cm^2^) was plotted against time; the trans-conjunctival flux (J, µg/cm^2^ h) was determined as the slope of the regression line at the steady state, while the apparent permeability coefficient (P_app_, cm/s) was calculated as:

P_app_ = J/Cd (1)

being Cd (µg/mL) the concentration of SVT in the donor chamber. Each condition was replicated at least 3 times.

#### 2.9.2. Trans-Scleral Permeation and Retention Experiments

For the evaluation of permeation across and retention inside the sclera (S) of SVT and SVA, Franz cells having a permeation area of 0.6 cm^2^ were chosen. In all the experiments, donor compartment was filled with a volume of 0.25 mL, while as receiving phase was used TPGS 0.5 mM in PBS pH 7.4, previously degassed and maintained at 37 °C and under magnetic stirring to avoid any boundary layer effect. The donor was alternatively 1 mg/mL SVT in TN_SVT_ (pH 6.78 ± 0.05), MN_SVT_ (pH 6.58 ± 0.02) and 1 mg/mL SVA saline solution (pH 6.75 ± 0.05). In control experiments, the donor chamber was filled with 0.25 mL of blank micellar suspension. After 48 h, the receiving phase was collected and analyzed; the donor was removed, the cell was dismantled, and the tissue was repeatedly washed using saline solution. Then, the permeation area was isolated by punching the sample with a 9 mm diameter die: resulting discs were soaked in extracting mixture (Section 2.8); SVT and SVA retained were quantified. SVT and SVA retention and permeation were also studied starting from micelles prepared by adding SVT previously dissolved in ethanol (see Supplementary Materials, Table S2).

Further experiments were carried out as described above using the trilayer SCh, having both pigmented and not pigmented choroid. 0.25 mL of 1 mg/mL TN_SVT_ in saline solution was used as donor. After 48 h, once the donor was removed, the cell was dismantled, and the tissue repeatedly washed using saline solution. Then, the permeation area was isolated by punching the sample with a 9 mm diameter die: S and Ch were separated and individually soaked in extracting mixture (Section 2.8) for the quantification of SVT and SVA retained. Each condition was replicated at least 3 times.

### 2.10. HPLC/UV-Vis Analysis

Quantification of SVT and SVA was simultaneously done using an HPLC/UV-Vis method, slightly modified from a previously published method [41]. In details, analysis was performed using an isocratic pump (Series 200, Perkin Elmer, Waltham, MA, USA) connected to an autosampler (Prostar 410, Varian, Leinì, Italy) and an UV–Vis detector (SPD-20A, Shimadzu, Kyoto, Japan), managed by Turbochrom workstation software (Perkin Elmer). As stationary phase a C18 column (Gemini 5 µm, 110Å, 250 × 3 mm; Phenomenex, Torrance, CA, USA) was chosen, while the mobile phase, eluted at 1.2 mL/min, consisted of 65% acetonitrile and 35% phosphate buffer (25 mM NaH_2_PO_4_, pH 4.5 with H_3_PO_4_). Temperature was set at 50 °C and UV absorbance was detected at 238 nm. Retention time was 2.5 min for SVA and 4.5 min for SVT. Method linearity was verified in the range 0.418–4.18 µg/mL and 4.18–83.6 µg/mL for SVA and 0.125–90 µg/mL for SVT. RSD% (relative standard deviation %) and RE% (relative error %) resulted lower than 5% and 10% respectively for all the concentration levels. The limit of quantification (LOQ) corresponds to the lowest concentration of linearity range.

### 2.11. Statistical Analysis

Data were reported as mean ± standard deviation, unless otherwise noted. The differences between values were assessed using Student’s t test and considered statistically significant when *p* < 0.05.

## 3. Results and Discussion

Statins represent the first line treatment in hypercholesterolemic disease and their effectiveness in the reduction of risk factors of cardiovascular issues is mainly due to the inhibition of HMGCoA reductase. This primary mechanism is also associated with the so-called pleiotropic effect, responsible for different favorable outcomes, such as anti-inflammatory and immunomodulatory effects. Due to different possible therapeutic effects statins might be introduced into the ocular framework, particularly for the treatment of AMD. In fact, in recent years, a growing number of scientific investigations highlighted the protectant role of statins on the posterior segment of the eye; nonetheless, to our knowledge, there are no specific studies addressing the efficacy of statins on the eye following ocular administration. Commonly, the treatment of the back of the eye is effectively achieved by invasive (i.e., intravitreal injections, implants) or less invasive (i.e., sub-conjunctival, periocular, via trans-scleral adsorption) approaches, also in combination with controlled release formulations, such as nanoparticles, liposomes, micelles, already in use or under investigation [42]. Against this background, the poorly soluble pro-drug simvastatin, a widely used systemic cholesterol lowering agent, was chosen as model compound, and proposed to address the ocular posterior segment delivered by polymeric micelles. Micelles, granting drug solubilization, ease of preparation, thermodynamic and kinetic stability, represent an emerging formulation for ocular delivery as demonstrated by the FDA approval of Cequa^®^ (SunPharma, Mumbai, India), a cyclosporine micellar product able to increase tear production in patients with keratoconjunctivitis sicca [43], and by the increasing number of papers addressing their development [29,44,45,46]. Among others, our group reported the preparation and characterization of polymeric micelles as colloidal carriers for poorly soluble compounds like cyclosporine, effective in severe dry eye syndrome, dexamethasone, often used for ocular inflammation, and econazole nitrate, for mycosis of the eye surface [32,38]. The same colloidal platform was chosen in this paper for the ocular delivery of SVT. As amphiphilic polymers, TPGS (hydrophobic portion: vitamin E; hydrophilic block: poly(ethylene oxide)—PEO) alone or mixed 1:1 with poloxamer 407 (P407; hydrophobic block: poly(propylene oxide)—PPO; hydrophilic blocks: poly(ethylene oxide)—PEO) were selected. TPGS is included in medical devices available in Europe (e.g., Visudrop™), while poloxamer 407 is approved for topical ophthalmic route by the Food and Drug Administration. In addition, TPGS and Poloxamer 407 mixed micelles appear non-irritant when evaluated by the chick embryo chorioallantoic membrane assay [32].

### 3.1. Micelles Characterization and Stability

TPGS (TN, 20 mM) and TPGS/P407 mixed micelles (MN, 10 mM:10 mM) showed viscosity values of 1.3 mPas and 33.2 mPas, respectively, and a Newtonian behavior. At the same time both micelles successfully enhanced the SVT solubility, that increased from 6.62 ± 0.91 µg/mL in saline solution (N) to 3690 ± 20 µg/mL in TN and 5580 ± 50 µg/mL in MN. As convenience, all experiments were performed keeping the SVT concentration in micelles at 1 mg/mL.

Table 1 reports the size of both blank and loaded micelles once prepared and after storage at room temperature for 48 h and 1 month. TPGS micelles were not influenced neither by drug-loading (TN vs. TN_SVT_), nor by storage length (time zero, 48 h, 1 month); mean size was always approximately 12 nm, with a very low PdI (around 0.1) indicating a narrow and almost monodisperse distribution.

On the contrary, mixed micelles, always presented three populations, with size less than 10 nm, 30 nm, and a third one with size larger than 100 nm that accounted for nearly 10% of the total intensity (Table 1). A similar pattern was already reported for poloxamer P407 alone at concentrations above 15% w/v, corresponding to approximately 12 mM [47]. Particularly, the smallest population, around 6 nm, was ascribed to single chains of the triblock copolymer, while the largest one, above 100 nm, represented micellar aggregates. Therefore, only the middle peak at approximately 30 nm represents (mixed) micelles; moreover, the presence of a single micellar population has been also demonstrated by small-angle X-ray scattering (SAXS) [38]. As for TN, also for mixed micelles SVT loading did not significantly affect the size. During the storage no substantial modification occurred, except for the larger population whose size and intensity weight increased over time, probably because of time dependent aggregation phenomena.

Concerning the drug stability, the amount of SVT encapsulated in TN_SVT_ micelles progressively decreased over time, particularly at the higher storage temperature. In fact, as shown in Table 2, after 1 month at 4 °C around 80% of initial SVT was detected, while the value drastically dropped to 30% when micelles were kept at 25 °C.

A diverse scenario was observed for MN_SVT_: after an initial loss of 20% in the first 48 h, SVT content remained almost stable until 1 month at 25 °C. Interestingly, the storage at 4 °C allowed for a complete recovery of SVT in MN_SVT_ micelles even after 1 month (Table 2).

Although, in general, micelles stabilize loaded drug(s), unlike other nanocarriers, they are characterized by a dynamic interchange among supramolecular assembled and free polymers chains, leading ultimately to a continuous micelles rearrangement [48]. The rate of this movement is strongly dependent on the nature and the concentration of polymer, the pH, the temperature, and affects in turn the dynamic equilibrium between incapsulated and non-incapsulated drug. The drug free in solution is more likely to face degradative processes, such as oxidation or hydrolysis, that lead to a progressive loss of cargo. In our micelles, while the hydrophilic corona consisted of only PEO (PEO100 for P407 and PEO23 for TPGS), the composition of the core was different. In fact, considering TPGS micelles, the core was exclusively represented by vitamin E, while in mixed micelles PPO was combined with polypropylene glycol blocks of P407. These structural variation account for a different core size [38] and probably for a different drug affinity and, ultimately, stability. In fact, a study on SVT-loaded polymeric micelles reported that the higher the hydrophobicity of the core, the more extended the stability of SVT [49]. Furthermore, other studies evidenced that short PEO chains, such as those of TPGS, account for low micellar stability because of a limited protection of the hydrophobic core towards aqueous environment [48,50]. At the same time, mixed micelles better stabilize cargo because the presence of two distinct polymers with different characteristics results in a less organized micellar core, in which the drug is trapped [49]. 

This dynamic process gives the chance to control over time the availability of simvastatin hydroxy acid (SVA), the simvastatin active form, which is dependent firstly on SVT release from micelles, and secondly on the SVT hydrolytic process regulated by both pH and temperature [31]. Once in contact with ocular membranes, SVT release and SVA formation are also mediated by esterase [51], ubiquitous enzymes, widely expressed in both human [52] and porcine [53] eye. In fact, esterase are able to directly hydrolyze TPGS, composing both TN and MN micelles, thus inducing SVT release and then its reversible hydrolysis to SVA [54]. Furthermore, TPGS hydrolysis leads to the formation of vitamin E and vitamin E succinate, as demonstrated also in vitro using porcine liver esterase [32]. Attempts to study in vitro the SVT release mediated by esterase from porcine liver were carried out. However, the simultaneous activity of esterase on different substrates associated to the temperature of 37 °C, per se favorable to hydrolysis, has made unattainable to discriminate against each individual contribution.

### 3.2. Trans-Conjunctival Studies

**Figure 2:**
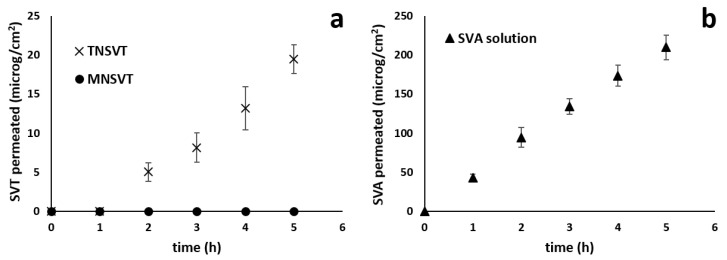
Trans-conjunctival permeation profiles of SVT from micelles (SVT 1 mg/mL; panel **a**) and SVA from 1 mg/mL saline solution (panel **b**).

The conjunctiva represents a thin transparent epithelial membrane, covering the entire ocular surface exposed to the environment, except for the cornea, as well as the internal side of eyelids. The conjunctiva, rich in blood and lymphatic vessels, expresses tight junctions, thus playing an important role in the eye protection. However, the ability of intact micelles to enter the corneal epithelium has been described [55], hence the diffusion of both TN_SVT_ and MN_SVT_ in the conjunctival epithelium cannot be excluded a priori. It was also reported that, once applied onto ocular surface, the drug might reach the posterior segment of the eye following the conjunctival-scleral route [56]. Therefore, to evaluate the feasibility of the topical administration of micellar formulation for the SVT delivery to the back of the eye, ex vivo trans-conjunctival permeation experiments were conducted. When testing a formulation that increases the solubility of a drug, usually a suspension of the insoluble compound is used as reference; unfortunately, in the present work this comparison was not possible since the high particle size of the SVT raw material (data not shown), lead to instant sedimentation on the tissue surface. Trans-conjunctival permeation profiles are shown in Figure 2.

Porcine conjunctiva modestly hindered SVT diffusion: after 5 h the amount permeated from TN_SVT_ resulted 19.51 ± 1.84 µg/cm^2^ (Figure 2a), and the apparent permeability coefficient, calculated using the Equation (1), corresponded to (1.58 ± 0.08) × 10^−6^ cm/s. However, SVT was not detected when MN_SVT_ were used (Figure 2a), suggesting that the larger size (25 nm vs. 12 nm; Table 1) as well as the different composition and consequently the different micellar structure, negatively impact on SVT permeation. Particularly, the smaller TN micelles are a monodisperse system, less viscous than MN mixed micelles, and consequently characterized by a higher mobility. Furthermore, the size, together with the hydrophobicity of the core, influence both thermodynamic and kinetic stability, thus having an impact on drug-micelle interaction [48], and ultimately on the drug release.

Last, SVT showed a higher thermodynamic activity in TN micelles than in mixed micelles. The best performance of TN_SVT_ micelles in the ex vivo condition could also be dependent on the tissue esterase. In fact, the enzymatic hydrolysis of vitamin E TPGS, was faster in TN micelles than in mixed micelles as previously demonstrated [32]. The lack of detectable SVA in the receiving chamber when TN_SVT_ and MN_SVT_ micelles were used indicates that SVT requires a time longer than five hours to be significantly hydrolyzed to active form, thus confirming a possible application for the sustained release.

When a SVA solution was used as donor, porcine conjunctiva did not hindered at all the diffusion (Figure 2b): in fact, the lag time, i.e., time required to reach the steady-state, was practically absent and the amount of SVA permeated after 5 h was 209.89 ± 15.56 µg/cm^2^, with the calculated apparent permeability coefficient of an order of magnitude higher than SVT from micelles, (1.07 ± 0.1) × 10^−5^ cm/s. From a physicochemical point of view, SVA shows a lipophilicity comparable with SVT (LogD_7_._4_ 1.8 vs. 1.6, respectively) and, at pH 7.4, is almost totally ionized. Therefore, a diversity of an order of magnitude between permeability coefficients has to be ascribed to the different formulations used (i.e., solution instead of micelles), that reflects in a different concentration gradient, considering that only the drug outside the micelles contribute to the gradient. Data also indicate that micelles composition highly affects drug trans-conjunctival transport (Figure 2a), as previously observed with other compounds. For example TPGS micelles allowed for a higher and statistically different conjunctival retention of econazole compared to poloxamer-TPGS mixed micelles [38].

### 3.3. Trans-Scleral Studies

Despite the high permeability demonstrated for SVT and SVA by conjunctiva, addressing the back of the eye by topical instillation presents several drawbacks. The main one is the systemic non-productive absorption (i.e., via conjunctiva and/or naso-lacrimal ducts), eventually responsible for not negligible systemic side effects. Therefore, the periocular administration followed by the trans-scleral absorption is a valuable alternative, being less invasive than intraocular and able to minimize the systemic effects. In fact, the formulation is injected in close contact with sclera to form a depot, from which the drug or even the nanocarrier is progressively released.

**Figure 3:**
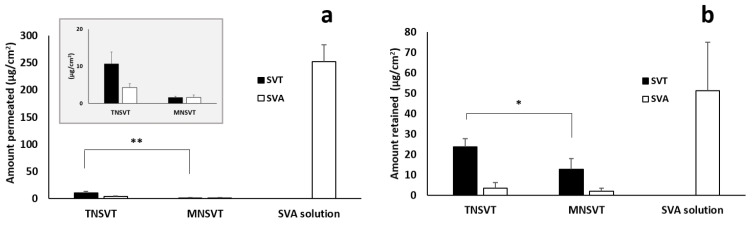
SVT (black) and SVA (white) permeated across (panel **a**) and retained (panel **b**) within porcine sclera after 48 h application of TN_SVT_, MN_SVT_ or SVA solution. In panel (**a**), an insert depicting the magnification of TN_SVT_ and MN_SVT_ is shown (statistically difference: * *p* < 0.05, ** *p* < 0.01).

Then the sclera, thanks to the high porosity (pore size ranging from 30 to 300 nm) ensures the diffusion of both low and high molecular weight compounds as well as nanocarriers [57]. Particularly, in the present work, the evaluation of SVT and SVA retention and permeation in 48h-lasting trans-scleral experiments was carried out, and data are presented in Table 3 and Figure 3.

SVT permeated and retained using TN_SVT_ resulted statistically higher (*p* < 0.01 and *p* = 0.04, respectively) than SVT permeated using MN_SVT_ micelles (Table 3; Figure 3), in agreement with the data obtained on the conjunctiva. This result, as discussed above (see Section 3.1), may be ascribed to a different overall structure/rigidity of the micellar assembly. Furthermore, smaller T micelles diffusion is favored in a such porous tissue like sclera and the retention of some intact micelles is expected [58]. On the contrary, no differences in terms of SVA formed from SVT within both tissue and receiving medium were observed (Table 3; Figure 3). When SVA is the primary permeant (SVA aqueous solution), the diffusion was practically not hindered thus resulting in a high amount of SVA both retained inside and diffused across sclera (Table 3; Figure 3).

Micelles, with respect to the SVA solution, sustained the release of SVT and consequently the SVA availability over time. TN_SVT_ showed a higher scleral retention than MN_SVT_ that may be ascribed to several factors such as the different size distribution (monodisperse vs. polydisperse), the different viscosity (less viscous vs. more viscous), and last the different polymer(s) composition (TPGS vs. TPGS:poloxamer). Polymers concentrations were selected not only on the basis of critical micellar concentration values [32], but also on their ocular tolerability [32,59], stability and formulation viscosity. Particularly, both micelles, thanks to their Newtonian behavior and a relatively low viscosity appear suitable for syringeability and injectability by 25 G or 27 G injection needles, required for periocular administration [60]. Furthermore, mixed polymeric micelles allowed for a slower release of SVT (Table 3), then progressively converted into the active form SVA. Despite a higher amount of SVT retained inside sclera from TPGS micelles, SVA detected was the same both within tissue and in the receiving medium (after 48 h), regardless of the use of TN_SVT_ or MN_SVT_ (Table 3). We may speculate that enzymatic and chemical hydrolysis in the tissue and receiving medium, requires a certain time to occur, thus minimizing the difference in term of SVA.

Reaching the target in trans-scleral delivery also means facing melanin, a polyanionic ubiquitous biopolymer, plentifully expressed in the eye, particularly inside choroid and RPE. Melanin may reduce the drug availability due to the high binding capacity exerted towards many compounds, especially those lipophilic and/or positively charged [61]. However, it is important to note that the interaction with melanin, is not always a negative aspect: in fact, when the binding is reversible, melanin may act as drug reservoir, allowing for a sustained release of the compound.

To our knowledge, currently no studies addressing the affinity of melanin for SVT are available, except for the demonstration of binding capacity of bacterial melanin for atorvastatin [62]. Despite the different chemical structure (atorvastatin vs. simvastatin) and the potentially relevant heterogeneity of melanin (bacterial vs. mammalian/human ocular), the relatively high SVT lipophilicity (LogD_7_._4_ 1.6 [33]), might support the hypothesis of a certain melanin affinity. As a consequence, a preliminary binding study was carried out with melanin from *Sepia officinalis* following a previously described protocol [40] demonstrating a not negligible affinity of SVT (data shown in Supplementary Material, Figure S1). However, the low SVT solubility associated to the relatively poor analytical sensitivity, limited the number of samples, and collected experimental data did not fit adsorption isotherm (either Langmuir, or Freundlich), thus preventing the calculation of binding parameters B_max_, the maximum binding of the ligand to melanin, and K_d_, the equilibrium dissociation constant for the binding complex ligand–melanin [63]. As alternative method, the validated model based on natural occurring pigmented and not pigmented choroids was selected [40]. With this aim, TN_SVT_ formulation was put in contact alternatively with a SCh trilayer (i.e., sclera, choroid, Bruch’s membrane), consist of choroid with and without melanin. After 48 h, the amount of SVT retained into choroid was not significantly influenced by the presence of melanin (Table 4), thus resulting in a negligible role of pigment on drug availability. Therefore, the retention of SVT within choroid was essentially ascribed to the lipophilicity of the membrane. 

## 4. Conclusions

Ophthalmic administration of statins may represent a new therapeutic approach for AMD, and, more in general, for inflammatory and immune diseases of the eye. The present research work focused the attention on the trans-scleral delivery as possible alternative administration approach by formulating SVT loaded micelles and studying ex vivo retention and diffusion behavior. Data collected demonstrate that polymeric micelles are an interesting platform for the sustained ocular administration of poorly soluble drugs, and could be administered either topically or, more efficiently, in the subconjunctival or periocular space. Particularly, even if their stability deserves to be increased, for instance by freeze-drying process, TPGS micelles appear best performing and, furthermore, represent a source of the antioxidant vitamin E, that could contribute on a side to increase the protectant effect of the formulation on the eye, and on the other side, to stabilize the drug. SVT significantly cumulated within sclera and, together with its active metabolite SVA, easily cross it.

Although promising, results deserve to be supported by specific in vivo studies in a relevant disease model. Furthermore, the demonstration of safety and efficacy of locally administered statins, particularly, formulated as polymeric micelles, as well as the definition of suitable therapeutic dose have to be duly obtained.

## Figures and Tables

**Table 1:** Size distribution with relative populations weight by intensity of blank and SVT loaded micelles measured at time zero and after 48 h and 1-month storage at room temperature (SVT concentration 1 mg/mL).

	Time Zero		48 h		1 Month	
Code ^a^	PdI	Size (nm and % Intensity)	PdI	Size (nm and % Intensity)	PdI	Size (nm and % Intensity)
**TN**	0.081	12.4 ± 3.6 (100)	0.024	11.7 ± 2.8 (100)	0.046	11.6 ± 3.0 (100)
**TN_SVT_**	0.142	13.2 ± 3.8 (100)	0.087	12.3 ± 3.8 (100)	0.032	11.7 ± 3.0 (100)
**MN**	0.26	5.0 ± 1.5 (49)	0.436	6.2 ± 2.5 (49.7)	0.201	4.9 ± 1.5 (41.6)
		27.8 ± 8.8 (42.7)		34.0 ± 15.1 (40.6)		31.0 ± 10.8 (51.6)
		165.1 ± 52.3 (8.3)		873.1 ± 480.8 (5.2)		158.8 ± 41.4 (6.9)
**MN_SVT_**	0.348	5.0 ± 1.2 (48.9)	0.367	5.3 ± 1.5 (42.1)	0.509	5.7 ± 1.6 (42.3)
		25.5 ± 6.7 (40.2)		25.0 ± 6.5 (40.7)		32.7 ± 10.8 (39.5)
		98.6 ± 17.8 (10.9)		636.6 ± 124.6 (16.7)		1443 ± 794.2 (18.2)

^a^ M= 10 mM TPGS:10 mM P407 micelles; N = saline solution as vehicle; SVT = simvastatin; T = 20 mM TPGS micelles.

**Table 2:** Amount of encapsulated SVT (as % of initial SVT, concentration 1 mg/mL) in micelles after 24 h, 48 h and 1 month at 4 and 25 °C.

	4 °C		25 °C	
Storage Time	TN_SVT_ (SVT%)	MN_SVT_ (SVT%)	TN_SVT_ (SVT%)	MN_SVT_ (SVT%)
**24 h**	96.9 ± 1.1	94.6 ± 6.8	92.7 ± 3.9	87.0 ± 13.9
**48 h**	91.8 ± 6.1	90.3 ± 12.1	86.7 ± 4.2	80.3 ± 8.8
**1 month**	79.5 ± 3.1	100.7 ± 0.3	32.4 ± 1.7	80.6 ± 4.1

M = 10mM TPGS:10 mM P407 micelles; N = saline solution as vehicle; SVT = simvastatin; T = 20 mM TPGS micelles.

**Table 3:** SVT and SVA permeated across and retained within porcine sclera after 48 h.

	Permeation		Retention	
**Code ^a^**	SVT (µg/cm^2^)	SVA (µg/cm^2^)	SVT (µg/cm^2^)	SVA (µg/cm^2^)
**TN_SVT_**	10.61 ± 3.27 **^b^**	4.26 ± 1.06	23.81 ± 3.88 **^c^**	3.54 ± 2.69
**MN_SVT_**	1.55 ± 0.36 **^b^**	1.61 ± 0.66	12.65 ± 5.39 **^c^**	1.95 ± 1.56
**SVA sol**	0 **^d^**	251.93 ± 31.23	0 **^d^**	51.42 ± 23.60

^a^ M= 10 mM TPGS:10 mM P407 micelles; N = saline solution as vehicle; SVT = simvastatin; T = 20 mM TPGS micelles. ^b^ *p* < 0.01; ^c^ *p* = 0.04; ^d^ under the LOQ.

**Table 4:** SVT and SVA retained in Sclera and Choroid after 48 h from TN_SVT_.

	Sclera		Choroid	
	SVT (µg/cm^2^)	SVA (µg/cm^2^)	SVT (µg/cm^2^)	SVA (µg/cm^2^)
**Pigmented Choroid**	30.07 ± 4.97	2.59 ± 0.74	1.01 ± 1.29	0 **^a^**
**Not pigmented Choroid**	30.15 ± 11.98	3.38 ± 1.05	1.06 ± 0.66	0 **^a^**

^a^ under the LOQ.

## Data Availability

Raw data are available upon request.

## Supplementary Materials

The following are available online at https://www.mdpi.com/article/10.3390/pharmaceutics13060855/s1, Figure S1: Binding curve of SVT to melanin from *Sepia officinalis* in saline solution, Table S1: Size distribution of micelles prepared by adding SVT as ethanolic solution. Data reported with relative populations weight by intensity and measured at time zero and after 48 h and 1-month storage at room temperature, Table S2: SVT and SVA permeated across and retained within porcine sclera after 48 h.
