# Supplementary material for: Preliminary Investigation on Simvastatin-Loaded Polymeric Micelles in View of the Treatment of the Back of the Eye
Source: Pharmaceutics. 2021 Jun 9;13(6):855. doi: 10.3390/pharmaceutics13060855 (PMC8230077; doi:10.3390/pharmaceutics13060855)
Supplement: Supplementary file 1 [file pharmaceutics-13-00855-s001.zip › pharmaceutics-1217605-SI.pdf]

pharmaceutics

MDPI

Supplementary Materials: Preliminary Investigation on Simvastatin-Loaded Polymeric Micelles in View of the Treatment of the Back of the Eye

Silvia Pescina, Fabio Sonvico, Adryana Clementino, Cristina Padula, Patrizia Santi and Sara Nicoli

Micelles prepared by adding SVT as ethanolic solution

As alternative loading method, 100 µl of 10 mg/ml SVT ethanolic solution was slowly dropped into 1 ml of TN or MN and kept under stirring (TxN$_{SVT}$, MxN$_{SVT}$ respectively). To allow for ethanol evaporation, solutions were stirred for two hours and left open overnight at room temperature. Following the same procedure, blank micellar formulations treated with ethanol (1:10 ratio) were prepared (TxN, MxN).

Table S1. Size distribution of micelles prepared by adding SVT as ethanolic solution. Data reported with relative populations weight by intensity and measured at time zero and after 48 hours and 1-month storage at room temperature.

	**Time zero**		**48 hours**		**1 month**	
**Code***	*PdI*	*Size (nm and % intensity)*	*PdI*	*Size (nm and % intensity)*	*PdI*	*Size (nm and % intensity)*
**TxN**	0.095	13.5 ± 4.5 (100)	0.027	13.9 ± 3.4 (100)	0.023	13.8 ± 3.1 (100)
**TxN$_{SVT}$**	0.126	13.2 ± 3.8 (100)	0.035	12.9 ± 3.2 (100)	0.025	13.2 ± 3.2 (100)
**MxN**	0.234	5.8 ± 1.9 (51.2)	0.481	7.1 ± 2.6 (49.7)	0.477	7.9 ± 3.8 (52.8)
		31.3 ± 10.4 (37.5)		37.2 ± 14.6 (34.6)		42.5 ± 19.7 (32.6)
		285.4 ± 85.4 (7.9)		1008 ± 615.2 (12.4)		1374 ± 852.1 (14.6)
**MxN$_{SVT}$**	0.229	5.8 ± 1.7 (51)	0.29	5.6 ± 1.9 (46.9)	0.334	5.7 ± 1.6 (42.5)
		29.2 ± 9.3 (40.3)		30.4 ± 9.4 (33.7)		32.1 ± 9.6 (33.8)
		168.7 ± 45.9 (8.7)		599.8 ± 161.2 (11.1)		761.2 ± 236 (14.8)

*M = 10mM TPGS:10mM P407 micelles; N = saline solution as vehicle; SVT = simvastatin; T = 20 mM TPGS micelles; x = ethanol as SVT solvent.

Table S2. SVT and SVA permeated across and retained within porcine sclera after 48 hours.

	**Permeation**		**Retention**	
**Code***	SVT (µg/cm²)	SVA (µg/cm²)	SVT (µg/cm²)	SVA (µg/cm²)
**TxN$_{SVT}$**	4.45 ± 4.25	3.76 ± 2.67	26.48 ± 9.73	5.35 ± 1.96
**MxN$_{SVT}$**	2.85 ± 0.57	5.89 ± 4.23	8.94 ± 2.34	2.17 ± 0.25

*M = 10mM TPGS:10mM P407 micelles; N = saline solution as vehicle; SVT = simvastatin; T = 20 mM TPGS micelles; x = ethanol as SVT solvent.

Melanin binding [1]

Melanin from *Sepia officinalis* (Sigma, St.-Louis, MO, USA) was weighted in a glass vial and directly dispersed with SVT solutions under magnetic stirring (melanin concentration range: 0.16-1 mg/ml; SVT concentration range: 1.07-8.83 µM; solvent: saline solution). Control solutions, without melanin, were also prepared. After incubation at room temperature for 1 h under magnetic stirring, the suspension was centrifuged at 12,000 rpm for 10 min. The supernatant was withdrawn and centrifuged again at 12,000 rpm for 10 min. Once separated, supernatant was diluted 1:3 with acetonitrile and analyzed for free SVT quantification, together with control solutions.

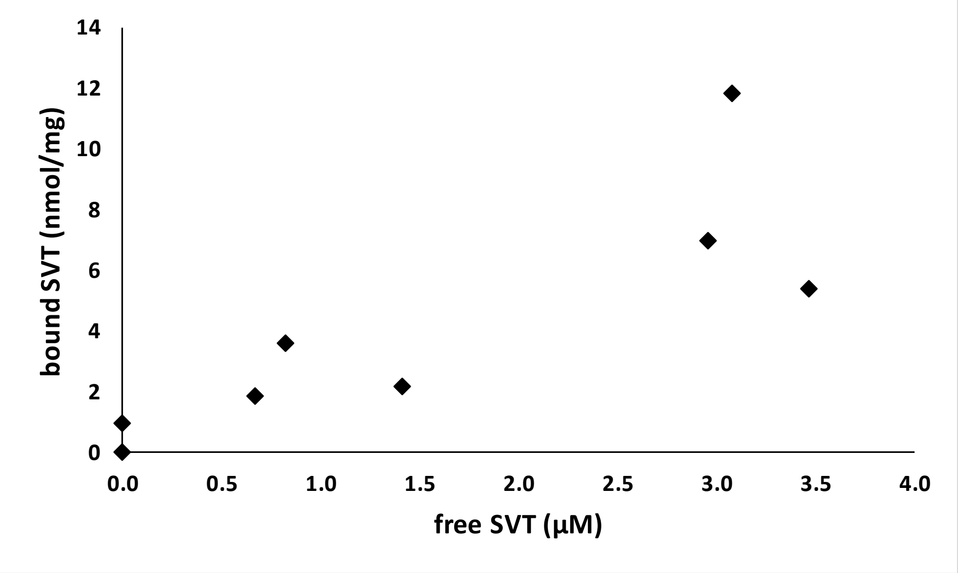

Figure S1. Binding curve of SVT to melanin from *Sepia officinalis* in saline solution.

The amount of SVT bound per mg of melanin is depicted as a function of the free SVT present in final solution.

References

1. S. Pescina, P. Santi, G. Ferrari, C. Padula, P. Cavallini, P. Govoni, S. Nicoli, Ex vivo models to evaluate the role of ocular melanin in trans-scleral drug delivery, *Eur J Pharm Sci*, **2012**, 475-483.
